# MTHFR Deficiency in Biological Siblings Diagnosed With Autism and Attention-Deficit Hyperactivity Disorder (ADHD): A Report of Two Cases

**DOI:** 10.7759/cureus.36294

**Published:** 2023-03-17

**Authors:** Samira Khan, Abeera Naeem

**Affiliations:** 1 Behavioral Health, WVU (West Virginia University) Berkeley Medical Center, Martinsburg, USA; 2 Behavioral Health and Psychiatry, West Virginia School of Osteopathic Medicine, Martinsburg, USA

**Keywords:** psychotropics, adhd, autism, genetic testing, mthfr c677t

## Abstract

Methylenetetrahydrofolate reductase is a critical enzyme that has been associated with several complex psychiatric mental health illnesses. The enzyme can be detected by bloodwork or a cheek swab and, once identified as lacking in individuals, can be treated by over-the-counter supplementation with folate. Due to a provider’s limited information and/or the cost to cover the test, the deficiency is not regularly tested for, and, therefore, is missed and not treated. There are very limited studies that demonstrate the benefits of supplements in conjunction with psychotropic medications. This study discusses the case of two biological siblings diagnosed with attention-deficit hyperactivity disorder and autism, who presented with this unique deficiency and had improvement of symptoms once starting the supplement with their traditional psychopharmacological treatment.

## Introduction

Recent data from 2019 to 2020 indicate that behaviors with conduct disorders are increasing in children [1]. Although conduct disorders can be seen on their own, they can be seen in children with autism spectrum disorder (ASD) and attention-deficit hyperactivity disorder (ADHD).

ASD is a developmental disability consisting of stereotypical behavior, limited communication, and impaired social skills. Subpopulations within the spectrum can present with various other co-occurrences of different severities. Each child with ASD presents differently and responds differently to treatment. As children with ASD grow and become young adults, they have difficulties interacting with their peers, managing themselves on their own, and understanding the behaviors expected of them in school or at work. They usually benefit from early intervention at home and school, which can assist them with activities of daily living, and most often have strategic plans in place to help them succeed [2].

ADHD is a neurobehavioral disorder characterized by symptoms of impulsivity, inattention, and hyperactivity [3]. These symptoms can present in any combination in the child. Boys tend to be diagnosed most often with all three symptoms while girls usually present with just inattentiveness or hyperactivity. Children diagnosed with ADHD are at higher risk for behavioral issues, other psychiatric disorders, difficulties with school, and problems at work. They have trouble focusing on given tasks, tend to be restless, will avoid tasks that require mental effort, make careless mistakes, and are frequently forgetful in daily activities [3]. These children usually require medications to get them through the school day. Many different medications exist for treating ADHD but there is no set algorithm that tells the prescriber which medication will work best. Usually, it’s a game of trial and error for determining which medication is best tolerated by the child and at which dose.

While both ADHD and ASD have completely different clinical presentations, it is known that they both have genetic links to their acquisition. Those genetic links are still being explored to this day; however, a common gene mutation has been found in both disorders, and it encourages us to further explore those genes and the treatment options that may be mutually beneficial to treating both conditions. Ultimately, treating psychiatric illnesses in well-rounded ways. The gene of interest that is associated with an increased risk of developing ASD or ADHD is the methylenetetrahydrofolate-reductase (MTHFR) 677C>T polymorphism, which impairs one-carbon (C1) metabolic pathway efficiency [4].

MTHFR is an enzyme that has been associated with several complex psychiatric mental health illnesses. It converts 5,10-methylenetetrahydrofolate to 5-methyltetrahydrofolate and participates in folate and homocysteine conversion correlated to DNA methylation [5-7]. Methionine is an amino acid acquired from foods and proteins that are used by our body to make essential proteins needed for daily functioning. When methionine is broken down, it is converted to Homocysteine, which remains in our bloodstream until it is broken down into cysteine and excreted in the urine [8]. The MTHFR enzyme works with vitamin B6, vitamin B12, and folate to recycle homocysteine in the blood and make more methionine for our body. Homocysteine accumulation is harmful to neurons and blood vessels, including the cerebral microvasculature, proposing a possible mechanism for the development of psychiatric illnesses like depression, dementia, autism, etc. [9].

To assess for MTHFR deficiency, providers can order a genetic test by cheek- swab or blood work. The genetic test analyzes how our genes may impact medication outcomes. It assesses genetic variations in DNA and provides information to physicians about how your body metabolizes certain medications that are given to treat psychiatric conditions such as depression, ADHD, and anxiety. Most insurances may not cover the tests unless the patient has a severe condition or has failed multiple treatment regimens [10].

Limited research has been done assessing the role of the MTHFR enzyme in psychiatric illness but from the few studies already performed, it has been found that the reduction of MTHFR activity or folate deficiency has been associated with an onset of several psychiatric diseases, schizophrenia, bipolar disorder, depression, autism, and ADHD [7-9]. The research that has been conducted thus far points to the information that DNA methylation and folate metabolism are important for maintaining mental health stability. Previous research illustrates the positive effects of L-methylfolate supplementation as an adjunctive treatment for certain mental illnesses [6]. However, there is limited research assessing how effective folate supplementation is in treating these mental illnesses and whether there is any benefit in patients with complex psychiatric comorbidities.

The following case series illustrates the journey of two biological siblings, a boy and a girl, with MTHFR deficiencies. They both went through an extensive trial of multiple medications leading up to their diagnosis. The cases below demonstrate the use of L-methylfolate in complex pediatric psychiatric cases with comorbid diagnoses.

## Case presentation

Case 1

The boy was four years old when he first came to the attention of behavioral health specialists. He was diagnosed with in-utero substance exposure, metopic craniosynostosis, global developmental delays, ASD with social-emotional reciprocity requiring substantial support (Level 2), restricted repetitive behavior requiring support (Level 1), with accompanying language impairment, without accompanying cognitive impairment, obstructive sleep apnea, and ADHD. He was placed in the foster care system secondary to neglect, of which the full details were unknown. His biological mother was using substances during the prenatal period, exposing him to cigarettes, methamphetamine, and opioids. As a result, he was born at 39 weeks gestation via emergency cesarean section due to late decelerations and prolonged fetal bradycardia. He had low birthweight and had significant meconium aspiration syndrome, which led to him being placed on high-flow intranasal oxygen, but subsequently needed intubation, and an oscillator. His post-natal care was unknown. He was adopted at 21 months and was observed to have delayed developmental milestones. He learned to walk at 21 months, received occupation and speech therapy through birth-to-three services, and transitioned to the head-start pre-K program at 2.5 years old.

His medication trial consisted of the following: clonidine, guanfacine, fluoxetine, long and short-acting methylphenidate, long and short-acting dexmethylphenidate, mirtazapine, prazosin, trazodone, viloxazine, risperidone, and amphetamine. The medications were discontinued after they either showed significant side effects or after they were given a full trial of optimizing the doses over a period of months and deemed ineffective. He completed his genetic test with a cheek swab when he was seven years old due to the delay in insurance approval, and he was found to have a severe MTHFR deficiency. The family started him on over-the-counter L-methylfolate 7.5 mg tablets once a day and noted great improvements in his behaviors two weeks later.

Case 2

The girl was three years old when she first started treatment for mental health concerns. She was diagnosed with in-utero substance exposure, fetal alcohol spectrum syndrome, global developmental delays, ASD requiring substantial support (Level 2), with accompanying language and cognitive impairment, and ADHD. She was born at 38 weeks gestation via C-section delivery and weighed 4 pounds and 5 ounces. She had aspirated on meconium-stained fluid during birth and had poor effort and tone upon delivery, resulting in her being intubated. She did well after intubation and was initially observed in the holding nursery but began exhibiting significant tachypnea and hypoxemia and was hypoglycemic on an initial heel stick. Thus, she was transferred to the neonatal intensive care unit (NICU) for further monitoring and treatment. She was extubated and weaned to room air the day after her delivery. She was monitored for neonatal abstinence syndrome. She was further monitored for substance withdrawal when she was placed in foster care at three months old and later was adopted at one year old. She had delayed developmental milestones and received speech, occupation, and physical therapy through birth-to-three. She transitioned into the head-start pre-K program at three years old. Her first neuropsychological evaluation was at age three in which she was diagnosed with global developmental delay and oppositional defiant disorder. For this, the family participated in parent-child interactive therapy (PCIT) when she was three to four years old for her behaviors. She only did well with PCIT when the therapist was present but was unsuccessful with it at home. When she was re-tested at age 6 she was diagnosed with ASD, level 2, and intellectual disabilities.

Her medication trials included: clonidine, guanfacine, risperidone, Depakote, lamotrigine, amphetamines, dextroamphetamine-amphetamine, methylphenidates, fluoxetine, and sertraline. The medications were stopped when they resulted in side effects or were deemed ineffective. She was five years old when she completed her genetic testing by cheek swab and was also found to have a severe MTHFR deficiency. The family started her on over-the-counter L-methylfolate 7.5 mg tablets once a day and noted great improvements in her behaviors two weeks later as well. The patient went from being defiant and oppositional to being able to be redirected at home and school, following through on instructions, and retaining information better than before.

## Discussion

Much research has been dedicated to learning about one of the most important enzymes involved in folate metabolism and pathways involving catecholamine neurotransmitters. The MTHFR C677T mutation is the most common MTHFR mutation [8]. In fact, the MTHFR 677CT mutation is found to be among the genes associated with the increased risk for autism in individuals and is further found in mothers of kids with autism [4,5]. This, by no means, is a direct correlation of MTHFR C677T to the diagnosis of ASD since ASD and its variability is due to multiple cofactors between genes and multiple environmental factors.

Decreased MTHFR activity, which results in decreased folate metabolism, can be seen in multiple psychiatric illnesses such as schizophrenia, bipolar disorder, depression, ASD, and ADHD. As discussed, research indicates the positive effects of l-methylfolate as adjunctive therapy for certain mental health illnesses [6]. Nevertheless, more research using behavioral scales studying the acute and chronic changes in mood and behaviors with and without the supplement in patients with behavioral health needs would be beneficial and be able to add to the medical knowledge within behavioral health. Studies have yet to show concrete evidence that folate supplements can be helpful in treating mental health illness, but they have demonstrated the importance of being able to detect the MTHFR deficiency in patients with and without mental health illnesses in order to develop a strategic treatment plan to help treat the individual with specific supplements such as folate, L-methylfolate, folic acid, and/or cobalamin [7].

## Conclusions

In summary, this article discusses two cases, that of biological siblings, with behavioral concerns, medication trials, and the discovery of MTHFR deficiency. Further research is needed to expand on this topic. Future steps include studying the long-term benefits of folate supplementation and identifying which psychiatric patients the supplement may or may not be effective with and how patients respond to traditional pharmacological treatment with and without the supplement. If positive effects are shown consistently, future work is needed to try and get Food and Drug Administration (FDA) support for this supplement so it can become a cost-effective treatment option for patients with an MTHFR deficiency and psychiatric comorbidities.
